# Assessment of vaccine information accuracy across large language models

**DOI:** 10.3389/fpubh.2026.1811462

**Published:** 2026-06-17

**Authors:** Julia Siebielec, Filip Raciborski

**Affiliations:** Department of Environmental Hazard Prevention, Allergology and Immunology, Warsaw Medical University, Warsaw, Poland

**Keywords:** ChatGPT, DeepSeek, generative artificial intelligence, Gemini, large language model, Copilot, vaccination, vaccine information

## Abstract

**Background:**

Use of Large Language Models (LLMs) as a health information source is on the rise. As the amount of people willing to self-diagnose with the tools’ help is increasing, verifying the correctness of generated responses becomes extremely important.

**Methods:**

Four LLMs (ChatGPT, Copilot, DeepSeek, Gemini) were chosen for the analysis. All models were asked 90 multiple-choice questions on the various topics surrounding vaccination, sourced from a government information website. Additionally, the LLMs were asked two open-ended questions regarding the vaccine information sources, as well as advantages and disadvantages of universal vaccination. Answers were analysed quantitatively and qualitatively. Data was collected in March 2026.

**Results:**

Biggest proportion of correct answers was achieved by Gemini (0.98), smallest one – by ChatGPT (0.86). Models’ results were found to be non-homogeneous (*p* = 0.0049). While answering open-ended questions, all LLMs were able to cite various, appropriate vaccine information sources. For advantages of vaccination all models pointed to herd immunity. Answers regarding disadvantages to vaccination all included the possibility of adverse reactions. The answers were judged to be factually correct, yet lacking in detail.

**Conclusion:**

Tested LLMs were able to identify correct option when presented with multiple choices and to prepare correct, yet vague information when asked an open-ended question. This points to a limited potential of LLMs serving as a vaccine information source, as the generated content, while factually correct, was not enough to be usable on its own.

## 1 Introduction

Large Language Models (LLMs) have gained notable popularity last couple of years, with July of 2025 seeing 10% of world’s adult population using ChatGPT (1). The technology is used in various ways, including information seeking (1), not excluding medicine-related queries (2), with as much as one in ten users asking artificial intelligence (AI) questions in that area (3). Some of the users go further, admitting willingness to self-diagnose with the tool’s help (4).

Use of LLMs as a health information source has already been a topic of research (5–8). Information reliability (5, 7), accuracy (5) and readability (6, 7) are subjects of interest in the analyses of generated content. Some of the studies evaluate content generated by one model (9), while others compare the work of multiple models (5–8, 10, 11). Monitoring the quality of generated content is important in the context of existing issues surrounding the models, such as algorithm limitations and the models’ hallucinations (12). Fast model progression and growing number of models available to the public cause research to become outdated quickly and create need for continuous testing of models’ abilities in providing reliable information (13).

In the area of AI-generated health information, topic of vaccination has already received some attention (14–16). Provision of accurate information regarding vaccines becomes significant with the context of current trends in vaccination levels; COVID-19 pandemic turned slowed vaccination rate gains of diphtheria-tetanus-pertussis, measles, polio and tuberculosis vaccines into declines (17), with numbers not recovering even in 2024 (18). To illustrate the issue better, in 2024 the marker of children immunization services’ quality (3rd dose of diphtheria-tetanus-pertussis vaccine) reached around 85% worldwide – which was still lower than pre-pandemic level of 86% (18). Additionally, at the same time around 20 million children globally received not enough or outright no vaccination (18). Forecasts of three mentioned vaccines for 2030 does not better the situation, with only 3rd dose of diphtheria-tetanus-pertussis vaccine reaching the global coverage goal planned in the Immunization Agenda 2030, only in the optimistic scenario (17). According to WHO, one of the factors impacting the vaccination rates is the issue of misinformation surrounding the topic (20). Director of the Department of Immunization, Vaccines and Biologicals at WHO points out the worrying trend in behaviour of people exposed to false information – relying on it even when provided with the accurate facts on the topic (20).

Given the significance of current trends of vaccination rates and the significance of providing the accurate vaccine information, this study focused on assessing accuracy of vaccine information generated by four chosen LLMs. In quantitative part of the analysis, we tested the models’ abilities of choosing the most correct information regarding vaccines from multiple presented options, in the form of multiple-choice tests in nine topics related to vaccination. For the qualitative part of the analysis, the models were asked two open-ended questions – the generated answers were evaluated in two dimensions: (1) correctness – that is, if the presented information is up to date and in accordance with scientific knowledge, and (2) completeness – that is, if the generated answer included information necessary for a layperson to gain basic understanding of the topic.

## 2 Materials and methods

Four freely available LLM engines were chosen for the analysis: OpenAI’s ChatGPT-5.3 (19), Microsoft’s Copilot, powered by ChatGPT-5.3 Instant (21), Google’s Gemini 3 (22) and High-Flyer’s Deepseek-V3.2 (23). The choice of particular engines was motivated by the popularity of each model – all four are included in top 7 of most popular LLMs: combined, they cover about 91% of traffic share, as of December 2025 (24).

To better mimic behaviour of laypeople, it was decided to use the most basic form of each chat: that is, a web version available without the account login was used, with all parameters set on default upon opening the chats. The engines were asked two open-ended questions and 90 multiple choice questions.

The open-ended questions were created by authors after subject literature review. The multiple choice questions were sourced from the vaccine information web portal created by institutions from Polish public health sector.

### 2.1 Quantitative analysis

Multiple choice questions for the analysis were sourced from *szczepienia.pzh.gov.pl* site, a free on-line portal created by National Institute of Public Health (PZH-PIB) (25) in collaboration with Polish Society of Vaccinology (PTWakc) (26). Creation of the portal was a part of WHO initiative of Vaccine Safety Network (VSN) (27). The site earned a WHO accreditation and is cited as a reliable source of vaccine information on the European Vaccine Information Portal (EVIP) (28). Portal was used as a question source to ensure the medical validity of the multiple-choice questions used during the analysis.

Questions used in the analysis concerned nine topics. The topics were chosen to cover broad spectrum of vaccination information that could be sought by a layperson: most topics concerned specific diseases that can be prevented by vaccination (rotaviruses, chickenpox, whooping cough, rabies); we also specifically added HPV as children vaccinations against this disease are a topic of public discourse in Poland (29); for the same reason the topic “COVID-19 vaccines for children” was added, as it also caused controversy (30). To assess the knowledge regarding the vaccination system in Poland, topics of adverse reaction to vaccination, mandatory and recommended vaccines [in Poland] and vaccine control were added. Together, nine topics of questions were included in the analysis; each included 10 multiple-choice questions. Material used in the analysis was chosen and used in a form as of May 2025.

Data gathering happened between 24 and 27 March, 2026: query sessions for quantitative analysis (multiple-choice questions) happened in following order: Gemini on 24th and 25th, Copilot – on 26th, Gemini and DeepSeek – on 27th. Data for qualitative analysis was gathered separately: all models had their query session on 27th. The web version of each model [available without the need to login into an account (31, 32, 52, 53)] was used to gather data. Each LLM was provided with a short prompt before questioning: “I will now ask you a question. Choose the most correct answer from options A, B, C or D. Choose only one answer”. Original prompt was written in Polish. No additional instruction was provided. A new chat was created for each question to minimise the possibility of earlier interaction impacting later responses.

A sample of multiple-choice question asked to the models, together with English translation, is presented in Table 1.

**Table 1:** Sample of multiple-choice questions used in the analysis.

Original questions used in the analysis	English translation
Jakie są przeciwwskazania do szczepień przeciw rotawirusom? a. Przebyte wgłobienie jelita. b. Nadwrażliwość na substancje zawarte w szczepionce. c. Występowanie biegunki lub wymiotów. d. Wszystkie odpowiedzi są prawidłowe.	*What are the contraindications for vaccination against rotaviruses?* *a. History of intussusception.* *b. Hypersensitivity to any substances contained in the vaccine.* *c. Occurrence of diarrhea or vomiting* *d. All answers are correct*
Kiedy najlepiej zaszczepić się przeciw HPV? a. Kiedy skończymy 18 lat b. W każdym wieku c. W wieku nastoletnim, najlepiej w wieku 9–14 lat d. W średnim wieku	*When is the best time to get vaccinated against HPV?* *a. When we turn 18.* *b. At every age* *c. During teenage years, best between 9–14 years of age* *d. In middle age*
Czym różnią się szczepienia obowiązkowe od zalecanych? a. Szczepienia obowiązkowe są bezpłatne. b. Szczepienia obowiązkowe dotyczą dzieci, a zalecane - dorosłych. c. Szczepienia obowiązkowe są ważne, a zalecane mniej ważne. d. Szczepienia obowiązkowe obejmują kwalifikację do szczepienia, natomiast zalecanych to nie dotyczy	*What is the difference between mandatory and recommended vaccinations?* *a. Mandatory vaccinations are free-of-charge.* *b. Mandatory vaccinations concern children, recommended – adults.* *c. Mandatory vaccinations are more important than recommended ones.* *d. Mandatory vaccinations involve qualifying examination, recommended do not.*

A rate of correct responses (0: no correct responses – 1: all responses were correct) was calculated for each LLM. Shapiro–Wilk test was performed to check the data distribution. A chi-square test of homogeneity was performed in search for significant differences between individual LLMs’ performance and two-sample Z-test for proportions was performed for each pair of LLMs to check for significant differences.

### 2.2 Qualitative analysis

In addition to multiple-choice questions, the LLM engines were asked two open-ended questions regarding vaccine information sources and advantages and disadvantages of universal vaccination. Original questions were written in Polish language. A new chat was created for each question. The answers were evaluated on the correctness (that is, if the presented information was on par with the current best practice and scientific knowledge) and completeness (that is, if the presented information was enough on its own for the user to gain a basic understanding of the topic) of included information.

The expectations for the models’ responses were following – as the models were told to treat the user as a layperson on a topic of vaccination, it was assumed that the response should include:

- Regarding the question about vaccination information sources – a list of multiple (more than 2) sources of vaccine information; the credible sources (e.g., government agencies, scientific journals); the exact, full names of specific sites/organizations, both local and international together with valid internet links leading to the part of the website specific for the topic of vaccine (that is, if, e.g., the model is suggesting Polish Ministry of Health as a vaccine information source, the included link should not lead the user to the main webpage of the Ministry, but to the webpage of the Ministry dedicated specifically to vaccines); the models should also inform user what type of the information they can find on the specific website, e.g., (if the source provide information on safety of the vaccines, the mandatory and recommended vaccines in Poland, the scheduling of vaccination appointments, etc.); the justification for inclusion of each source recommended by the model (e.g., “this site was included in the answer because it leads to the organisation responsible for guidelines which vaccines should be recommended and which should be mandatory”); the sources the models used to compile the answer (valid internet links to specific websites).
- Regarding the question about advantages and disadvantages of universal vaccination – a list of scientifically correct advantages and disadvantages of universal vaccination, with each argument backed by a justification; statistics simple enough to be understood by a person without expert knowledge, yet specific enough to paint an accurate picture of the scale (e.g., if the model cites the adverse events of vaccination as a disadvantage of universal vaccination, it should include statistics regarding how often such events happen yearly in Poland, for how many administered doses, etc.); the sources the models used to compile the answer (valid internet links to specific websites).

The list of evaluation criteria in correctness and completeness categories is presented in the Table 2. The criteria were established before the commencement of the evaluation. Two reviewers (JS & AS) completed the evaluation independently; it was agreed to rate each criterium fulfilment with “Yes,” “No” or “Partially”; short comments and justifications of judgement were also allowed. In case of significant differences of opinion, a discussion was held to reach a consensus. Table 2 includes all the answers in this situation [answer of JS, answer of AS and the consensus answer (“Final verdict” with a short justification if needed)].

**Table 2:** Evaluation criteria used for open-ended question responses.

Question	Category	Criterium
Question 1	Correctness	Does the LLM present user with credible sources of information in its answer?
		Are the provided sources verifiable (does LLM provided name of the organisation, link to the website)?
		Does the LLM provide source(s) of produced information?
	Completeness	How many sources does the LLM provide?
		Does the LLM provide description of what can be found at each provided choice?
		Does the LLM provide the justification for inclusion of each provided source?
Question 2	Correctness	Does LLM provide sources of produced information?
		Are the provided advantages and disadvantages consistent with current scientific knowledge?
	Completeness	Does LLM provide justification for the choice of advantages and disadvantages included in the answer?
		Does LLM provide statistics in its answer to back the presented argument?

Before being asked each question, LLM was presented with a short prompt, originally in Polish: “Prepare short (up to 200 words) answer to the asked question. Prepare the answer in the form of the list. The answer should be informative and concise. Use everyday language – assume that the user is a layperson on the topic.”

Questions, translated into English, were following:

- What sources should be used to access vaccine information?
- List the biggest advantages and disadvantages of universal vaccine mandates.

A new chat was created for each question to minimise the possibility of previous interactions influencing the later response.

## 3 Results

### 3.1 Quantitative analysis

The proportion of correct responses ranged from 0.98 to 0.86. The biggest proportion of correct answers belonged to Gemini (0.98 [95% CI: 0.95; 1.01]), followed closely by DeepSeek (0.97 [95% CI: 0.94; 1.01]) and Copilot (0.92 [95% CI: 0.79; 0.93]). ChatGPT presented the smallest proportion of correct answers in the test (0.86 [95% CI: 0.79; 0.93]). The mean result was 0.93 (95% [CI: 0.88; 0.98]) with standard deviation of 0.06 and standard error of 0,028.

The results of LLMs’ were found to be distributed normally (*p* = 0.418). Chi-square test of homogeneity found the results to be not homogeneous (*p* = 0.0049).

To be able to meaningfully compare the results between LLMs, each model was paired with the others and a two-sample Z-test for proportion was performed. Statistically significant differences were found only between Gemini and ChatGPT (z = 2.97; *p* = 0.003) and Copilot and DeepSeek (*z* = −2.62; *p* = 0.008). Results of all performed two sample Z-tests for proportions are presented in Table 3. As four out of six tested pairs were not found to be statistically significant, the models were concluded to perform comparably to one another, despite the raw proportions being different among the models.

**Table 3:** Two sample Z-tests for proportion results.

Model	Correct answers	Sample size	Proportion	Z-statistic	*p*-value
Gemini vs Copilot					
Gemini	88	90	0.98	1.71	0.08
Copilot	83	90	0.92		
Gemini vs ChatGPT					
Gemini	88	90	0.98	2.97	0.003*
ChatGPT	77	90	0.86		
Gemini vs DeepSeek					
Gemini	88	90	0.98	0.45	0.650
DeepSeek	87	90	0.97		
Copilot vs ChatGPT					
Copilot	83	90	0.92	1.42	0.154
ChatGPT	77	90	0.86		
Copilot vs DeepSeek					
Copilot	83	90	0.92	0.94	0.193
DeepSeek	87	90	0.97		
ChatGPT vs DeepSeek					
ChatGPT	77	90	0.86	−2.62	0.008*
DeepSeek	87	90	0.97		

*Statistically significant.

Incorrect answers were present in all topics with various amounts. The summary of incorrect answers of each LLM is presented in Table 4.

**Table 4:** Incorrect answers summary for each LLM.

Topic	ChatGPT	Gemini	Copilot	Deepseek	Sum
Rotaviruses			1		1
HPV	1				1
Chickenpox	1	1	1	1	4
Whooping cough	1				1
Rabies	2				2
COVID-19 vaccine & children	2		2	1	5
Adverse reaction to vaccination	2			1	3
Mandatory and recommended vaccines [in Poland]	2	1	2		5
Vaccine control	2		1		3
Sum	13	2	7	3	25

In some instances a question was answered incorrectly by multiple models: all LLMs were unable to choose correct description of the chickenpox vaccine. ChatGPT and Copilot also incorrectly guessed the rationale behind vaccinating children against COVID-19, as well as were not able to say whether or not mRNA parts of COVID-19 vaccine cause long-term adverse reactions; questions about changes in Polish vaccination schedule in recent years and control level of vaccines in Poland also caused both models difficulty.

### 3.2 Qualitative analysis

The LLMs’ answers were evaluated on the basis of correctness and completeness with criteria described in Table 1, established prior to the evaluation. The evaluation was conducted by two reviewers independently; in case of significant disagreements, discussions were held to reach a consensus.

#### 3.2.1 Vaccine information sources

All LLMs provided multiple sources of information. Number of sources varied from 6 (Gemini) to 11 (DeepSeek).

All LLMs mentioned WHO as a credible source of information; all also provided a mix of international and local health organisations. The most commonly mentioned sources were: Ministry of Health and medical personnel. CDC was mentioned two times, same as ECDC. Scientific sources were either cited in form of entire databases (Copilot, Gemini) or descriptively as “peer-reviewed scientific publications in reputable medical journals” (DeepSeek). ChatGPT additionally included “reliable medical information” but did not specify further.

Copilot, Gemini and ChatGPT included a short justification for inclusion of each source, citing either relevance to the topic of vaccination or type of information available at each source.

Summary of vaccination information sources provided by each LLM is presented in the Table 5.

**Table 5:** Summary of vaccination information sources.

ChatGPT	Copilot	Gemini	DeepSeek
Ministry of Health	Ministry of Health	szczepienia.pzh.gov.pl*	WHO
National Institute of Public Health	Chief Sanitary Inspectorate	WHO	ECDC
WHO	Szczepienia.info**	ECDC	National Institute of Public Health
General practitioner	WHO	General practitioner	Chief Sanitary Inspectorate
Paediatrician	CDC	Summary of Product Characteristics	Ministry of Health
CDC	Pubmed	Pubmed	szczepienia.info*
Pharmacists	Medical personnel: doctor, nurse, pharmacist		General practitioner
“Reliable medical websites”			Paediatrician
Medical documentation			Infectious disease specialist
			Vaccination centre staff
			“Peer-reviewed scientific publications in reputable medical journals”

*Online portal of Polish National Institute of Public Health; **online health information platform created by Polish Ministry of Health.

Additionally, Gemini and Deepseek advised users against using social media and internet discussion sites during information search in their responses, citing lack of content verification as a main reason to avoid such sites. At the end of the answer, Copilot and Gemini prompted user to ask further questions about specific issue related to vaccination.

The LLMs’ answers were evaluated on the basis of correctness and completeness with criteria described in Table 1, established prior to the evaluation. Summary of evaluation results is presented below:

- Correctness assessment: The LLMs managed to provide mostly credible sources of vaccine information with the exception of two descriptive sources (Copilot’s “Reliable medical websites” and DeepSeek’s “Peer-reviewed scientific publications in reputable medical journals” – there was no exact source provided, only a description of what the user should look for). The answers lacked necessary details, which lowered their usability, as no LLM provided direct links to mentioned organizations/institutions and only Copilot provided a source it used to compile the list. The provided links were checked to be functional and leading to intended information: the model used a mix of official sources, such as sites of government bodies and healthcare providers, and unofficial ones, such as news portals focused on health-related topics.
- Completeness assessment: The number of provided sources varied among the LLMs (ranging from 6 to 10); all LLMs presented a balanced variety of sources by inclusion of both local and international sources; all included WHO and either American or European Centre for Disease Control (CDC/ECDC). The answers were lacking in content descriptions and choice justifications – the expectation was that LLMs would include a description of what kind of information a user can expect while using each source (e.g., “this source includes information regarding Polish vaccination calendar”), as well as an explanation why a particular source was included in the answer – e.g., that the link leads to the official site of an organisation responsible for vaccine recommendations in Poland and the information found on the site is sure to be up-to-date and correct.
- Final assessment: The produced answers, while including true information and presenting a broad spectrum of vaccine information sources, were not detailed enough to be fully usable on its own. In the situation of actual vaccine information seeking the user would need to ask additional question to find out which of provided sources would be most suitable to their individual needs.

The full evaluation is presented in the Table 6.

**Table 6:** Open-ended questions: answer evaluation.

Category	Critetium	ChatGPT		Copilot		Gemini		DeepSeek	
Question 1									
		Rev. 1	Rev. 2	Rev. 1	Rev. 2	Rev. 1	Rev. 2	Rev. 1	Rev. 2
Correctness	Does the LLM present user with credible sources of information in its answer?	Yes	Yes	Partially – for one source only description was included (“Reliable medical websites”)	Yes	Yes	Yes – without much detail	Partially – for one source only description was included (“Peer-reviewed scientific publications in reputable medical journals”)	Yes
	Are the provided sources verifiable (does LLM provided name of the organisation, link to the website)?	Partially – only names, no direct links	Partially – only organisation names, without direct links	Partially – only names, no direct links	Partially – only organisation names, without direct links	Partially – only names, no direct links	Partially – only organisation names, without direct links	Partially – only names, no direct links	Partially – only organisation names, without direct links
	Does the LLM provide source(s) of produced information?	No	No	Yes	No	No	No	No	No
				Final verdict: Yes*					
Completeness	How many sources does the LLM provide?	10		9		6		10	
	Does the LLM provide description of what can be found at each provided choice?	No	Yes	Partially – only for part of the presented arguments	No	Partially – only for part of the presented arguments	No	No	No
		Final verdict: Partially – the descriptions give a very general idea of contents							
	Does the LLM provide the justification for inclusion of each provided source?	Yes	Yes – without much detail	Partially – only for part of the presented arguments	Yes – without much detail	Yes	Yes – without much detail	No	No
Question 2									
		Rev. 1	Rev. 2	Rev. 1	Rev. 2	Rev. 1	Rev. 2	Rev. 1	Rev. 2
Correctness	Does LLM provide sources of produced information?	No	No	Yes	No	No	No	No	No
				Final verdict: Yes*					
	Are the provided advantages and disadvantages consistent with current scientific knowledge?	Yes	Yes	Yes	Yes	Yes	Yes	Yes	Yes
Completeness	Does LLM provide justification for the choice of advantages and disadvantages included in the answer?	No	Yes – without much detail	Partially – only for part of the presented arguments	Yes – without much detail	Yes	Yes – without much detail	Yes	Yes – without much detail
		Final verdict: Partially – the justifications are vague and short							
	Does LLM provide statistics in its answer to back the presented argument?	No	No	No	No	No	No	Partially – only for one example	No
								Final verdict: No – the included statistic is of poor value and only includes an estimation	

Difference in the evaluation between these particular questions was due to the file error.

#### 3.2.2 Advantages and disadvantages of universal vaccination

Three LLMs provided a list of advantages and disadvantages of universal vaccination without any comment; DeepSeek has included a short paragraph near the end (“scientific context”) emphasizing the fact that the benefits of universal vaccinations far outweigh the possible risks.

With advantages of universal vaccination, LLMs focused mostly on patient benefits, though the effects on healthcare systems were also included. Herd immunity was mentioned by all four LLMs, eradication (or near-eradication) of certain diseases – by three. DeepSeek included a perspective beyond health – economic benefits of vaccination (namely smaller costs of disease and complications treatment, less disease-associated absences and higher productivity of society).

Summary of advantages cited by each LLM is presented in Table 7.

**Table 7:** Summary of LLM answers - advantages of universal vaccination.

ChatGPT	Copilot	Gemini	DeepSeek
Protection against dangerous diseases	Protection against dangerous diseases	Individual protection	Control and eradication of diseases
Reduced risk of severe illness and complications	Reduced risk of complications and death	Herd immunity	Individual protection
Herd immunity	Herd immunity	Eradication of diseases	Herd immunity
Protect those who cannot be vaccinated (very young children, elders)	One of the cheapest and most effective methods of public health protection	Save time and money	Economic benefits
Eradication and near-eradication of certain diseases	High level of security		Reduction of antibiotic resistance
Well-researched and continuously monitored			Travel safety and global health

For disadvantages of vaccinations, all LLMs mentioned adverse reactions; two (ChatGPT, Gemini) emphasized the rarity of such event occurring. Lack of 100% effectiveness guarantee and need for booster doses were mentioned three times each. Both ChatGPT and DeepSeek mentioned uncertainty surrounding the vaccines caused by the lack of accurate information on the topic. From the health system perspective, logistic issues were mentioned by three LLMs (ChatGPT, Copilot, DeepSeek).

Summary of disadvantages cited by each LLM is presented in the Table 8.

**Table 8:** Summary of LLM answers - disadvantages of universal vaccination.

ChatGPT	Copilot	Gemini	DeepSeek
Can cause mild side effects	Can cause rare, mild side effects	Adverse reactions	Adverse reactions
Can (rarely) cause more severe adverse reactions	Can (rarely) cause allergic reactions	Lack of 100% effectiveness guarantee	Costs and logistics
Lack of 100% effectiveness guarantee	Lack of 100% effectiveness guarantee	Need for repeated vaccination in some instances (boosters)	Fear and discomfort
Need for repeated vaccination in some instances (boosters)	Need good health system organisation (to uphold high vaccination rates)	Stress and discomfort	Need for repeated vaccination in some instances (boosters)
Uncertainty surrounding the safety (due to misinformation)			Rare contraindications
Logistic issues surrounding the organisation of vaccinations (time and availability)			Disinformation and lack of trust

The LLMs’ answers were evaluated on the basis of correctness and completeness with criteria described in Table 1, established prior to the evaluation. Summary of evaluation is presented below:

- Correctness assessment: all LLMs provided correct information; however, only one of them included sources used during generated the answer, which unnecessarily complicates the potential fact-checking process.
- Completeness assessment: the LLMs’ choice justifications, while present in the answers, were deemed not detailed enough – models did not explain their reasoning in a way that would be understandable for a layperson, by, e.g., using specialized terms such as “herd immunity” or “adverse events” without providing their definition; as the prompt asked the models to treat the user as not an expert on the topic, that was an expectation. Additionally, the models in majority did not include any statistics in their responses; the one model that did include one did so with an estimation, not an actual value, which could give a general idea of the scale, but not the actual information.
- Final assessment: while the LLMs generated correct information, it is also vague and not backed by numbers, which reduces the answers’ usability. Lacking list of sources used to prepare answers exacerbates the problem by hindering any attempts to check the credibility of presented information.

The full evaluation is presented in the Table 6.

## 4 Discussion

### 4.1 Interpretation

The results achieved by all models in the quantitative analysis (proportion of correct answers above 0.85) may be a result of information recall rather than their analytical abilities. The site the questions were sourced from is publicly available – there is a non-zero possibility that it was included in the data the models were trained on. The highest amount of mistakes committed in the COVID-19 vaccines & children section may be attributed to considerable amount of misinformation surrounding the COVID-19 in Polish online spaces (33, 34), which also could have been used in the process of training the models. As for the mandatory and recommended vaccines in Poland, the number of mistakes may be a result of ever-changing nature of the guidelines (they are revised yearly (35)) – possibly, LLMs were also trained on the sites containing both revised and old information and had an issue with differentiating between archival and current recommendations. Instances of Copilot and ChatGPT committing the same mistakes might be attributed to the fact that version of Copilot used in this analysis was powered by ChatGPT (21). As the statistical significance was found in only two pairs of models (ChatGPT-Gemini and Copilot-DeepSeek) out of six tested, we can conclude the models operate on a comparable level.

As for the assessment of answers to open-ended questions presented in the qualitative analysis, the reviewers rated the models moderately: the answers were factually correct, yet not detailed enough to be deemed usable on its own; additionally, there was a repeating issue with lack of source provision, which also lowered the quality of generated content.

### 4.2 Generalisability

The LLMs’ performance is being tested in multiple-choice-based examinations rather often (36–41). Questions are sourced from various types of examinations used in medical schools (36, 38, 39) or self-assessment tools (41) and study materials (40); most of these analyses cover multiple areas of medicine (37, 38, 40). Our quantitative analysis concluded that the performance of the ChatGPT, Copilot, Gemini and DeepSeek on questions regarding vaccines in Polish language was on the same level, regardless of used model, with the mean result of 0.93. This result is better than by the result achieved by ChatGPT-3.5 in public health-related questions from Polish Medical Final Examination (0.76) (38) and in Polish national specialty examination in radiology (0.52) (42). Similarly, ChatGPT-4.0 achieved around proportion of correct answers of around 0.7 on nephrology specialty exam of Poland (43). In a similar analysis regarding the topic of vaccines specifically, ChatGPT managed to achieve a similar result (around 0.9) while being tested on questions regarding COVID-19 vaccine; notably, the questions were in English and the amount of questions was noticeably smaller than in our analysis (44). Although multiple-choice test is a popular method of LLMs’ examination, there are concerns regarding its ability to truly reflect the understanding of medical concepts by LLMs (45).

Qualitative analysis of LLMs’ answers for open-ended questions in our analysis resulted in a moderate score of correct, but not detailed enough. In a study examining ChatGPT-3.5’s and Chat-GPT-4’s responses to WHO’s myths and misconceptions about vaccines, the generated content was judged to be easily understandable and in majority accurate, with ChatGPT-4’s versions of answers being more correct, clear and exhaustive (46). The accuracy of LLMs’ answers was also observed in a study comparing the abilities of ChatGPT-4o, Copilot and Gemini Flash 2.0 in tackling questions regarding childhood vaccination; there were statistically significant differences between models’ performances: all models generated accurate content, with ChatGPT’s responses being ranked the highest, followed by Copilot and Gemini (47). Lack of detail in answers, observed in our analysis, has been noted by other researchers in a qualitative analysis of HPV vaccine information generated by ChatGPT, Claude, DeepSeek and Docus (48).

The role language in the analysis cannot be underestimated – in the analysis comparing ChatGPT-4’s responses regarding vaccination in English and Spanish, Spanish responses rated slightly lower in terms of understandability than responses in English, but the difference was not significant (49). Spanish responses were rated significantly higher in terms of readability (49). The accuracy was high, similarly rated in both languages (49). In an analysis form comparing the performance of ChatGPT-4o, Llama 3.1-405b-Instruct, Llama 3.3-70b-Instruct, Claude-Sonnet-3.7-20250219 and DeepSeek V3 regarding various specialities of medicine, including public health, in German, French and Italian, the models were most accurate in German, with no significant difference to French version, but significantly lower performance in Italian one; importantly, Claude and ChatGPT had highest accuracy consistently, regardless of the language (41). Our analysis did not include the comparison between different languages, focusing on Polish exclusively; yet, while interpreting the results it is important to consider that information regarding vaccines may have different densities in different languages, which may impact the abilities of the models to answer questions accurately.

The issue of not providing citations observed in our analysis has been noted by other researchers as well – in one study testing ChatGPT-3.5 and ChatGPT-4, only 18% of references was provided to science-related questions without explicit request of the user; more so, about 70% of provided references included a link, out of which 27% was non-functioning and 0.6% was a hallucination of the model (50).

In our analysis, the provided references lead to valid websites containing information supporting the claims presented by the model. In another study, focused on medical references, two versions of ChatGPT-4o (RAG & API), Claude v2.1, Minstral Medium and Gemini Pro were compared – even for the most advanced model (ChatGPT-4 RAG) about 40% of generated responses was not supported by provided references (51). For the next best result in the study – ChatGPT-4 API – this percentage falls to below 30% (51). When the references with internet are provided, their ability to return an actual website varies greatly between models (51).

### 4.3 Study limitations

Analysis presented in this article had several limitations:

- Fast advancement of LLMs – our results are a reflection of abilities of a very particular version of each model (that is, the newest one available free of charge at the time of data collection). It is possible that new versions of tested models will be made available to the public before publication of this manuscript.
- The choice of LLMs – the selection of models used in this analysis was made based on the popularity rankings of the LLMs, without regard to differences between models, e.g., update frequency, architecture or training data, which introduces unnecessary variable into the analysis.
- Use of web login-free version of the models – the decision to use free, web-based versions of LLMs, while better mimicking the behaviour of the layperson, did not allow to control multiple prompt parameters, e.g., dynamic retrieval mechanisms, prompt temperature or memory settings. This lack of standardisation limits both the comparability of the models and the replicability of the findings, lowering the methodological quality of the analysis. Additionally, during data collection we did not collect information on several variables, such as browser conditions, session persistence, caching effects, or temporal variability in model outputs. The data was collected in span of 2 days, which also adds an uncontrolled variable to the analysis.
- Single-theme evaluation – the questions used in the analysis, although varying in topics, all orbited around one theme – vaccination. All multiple-choice questions were also sourced from one freely available website – it is possible data contamination might have occurred, as the entire site, quizzes including, might have been scraped and used to train models included in the analysis. It is thus entirely possible the high scores achieved by all models may be thanks to recalling the already known information rather than analytic and reasoning skills of the models.
- One source of multiple-choice questions – the use of only one portal as a source for all multiple-choice questions additionally limits the generalisability of the findings
- Nature of multiple-choice questions – another limitation is that the questions used in the quantitative analysis may also not accurately reflect typical information-seeking behaviour of patients, being closer in form to academic evaluations. There are also concerns if this type of questions is an accurate tool to reflect the understanding of medical concepts by LLMs (45).
- Data density – vaccination as a health topic has the optimal situation of abduance of data on the topic being freely available on-line and that data being used to train models – this may not be occurring in other languages and more niche health-related topics, such as rare diseases. In this instance, the models’ abilities to answer questions regarding the topic would be lower.
- Non-validated tool for qualitative evaluation – the evaluation tool was developed for this particular analysis and was not validated prior to use, the evaluation is significantly burdened by the subjectivity of the reviewers, which introduces significant methodological weakness into the analysis.

## 5 Conclusion

LLMs tested in our analysis proved to be able to identify correct option when presented with multiple choices and to prepare correct, yet not nearly detailed enough information regarding vaccines when asked an open-ended question. This points to a limited potential of LLMs serving as a vaccine information source, as the generated content was not enough to be usable on its own. Providing general public with educational materials regarding the limitations of LLMs in the role of vaccine information source may raise public awareness and lead to exercise caution while engaging with vaccine-related content generated by the models.

## Data Availability

The raw data supporting the conclusions of this article will be made available by the authors, without undue reservation.
